# Zebrafish (*Danio rerio*) behavioral laterality predicts increased short-term avoidance memory but not stress-reactivity responses

**DOI:** 10.1007/s10071-019-01296-9

**Published:** 2019-07-24

**Authors:** Barbara D. Fontana, Madeleine Cleal, James M. Clay, Matthew O. Parker

**Affiliations:** 1 Brain and Behaviour Laboratory, School of Pharmacy and Biomedical Sciences, University of Portsmouth, White Swan Road, Portsmouth, PO1 2DT UK; 2 The International Zebrafish Neuroscience Research Consortium (ZNRC), 309 Palmer Court, Slidell, LA 70458 USA; 3 Department of Psychology, University of Portsmouth, King Henry I Street, Portsmouth, PO1 2DY UK

**Keywords:** Anxiety, Behavioral asymmetry, Free movement pattern Y-maze, Pavlovian fear conditioning, Laterality bias

## Abstract

Once considered a uniquely human attribute, behavioral laterality has proven to be ubiquitous among non-human animals, and is associated with several neurophenotypes in rodents and fishes. Zebrafish (*Danio rerio*) is a versatile vertebrate model system widely used in translational neuropsychiatric research owing to their highly conserved genetic homology, well-characterized physiological responses, and extensive behavioral repertoire. Although spontaneous left- and right-biased responses, and associated behavioral domains (e.g., stress reactivity, aggression, and learning), have previously been observed in other teleost species, no information relating to whether spontaneous motor left–right-bias responses of zebrafish predicts other behavioral domains has been described. Thus, we aimed to investigate the existence and incidence of natural left–right bias in adult zebrafish, exploiting an unconditioned continuous free movement pattern (FMP) Y-maze task, and to explore the relationship of biasedness on performance within different behavioral domains. This included learning about threat cues in a Pavlovian fear conditioning test, and locomotion and anxiety-related behavior in the novel tank diving test. Although laterality did not change locomotion or anxiety-related behaviors, we found that biased animals displayed a different search strategy in the Y-maze, making them easily discernable from their unbiased counterparts, and increased learning associated to fear cues. In conclusion, we showed, for the first time, that zebrafish exhibit a natural manifestation of motor behavioral lateralization which can influence aversive learning responses.

## Introduction

Lateralization of brain and behavior is the apparent predisposition towards side bias, which often manifests in terms of motor output, such as handedness. In humans, functional laterality of different brain regions is important in both language and cognition (Hull and Vaid 2007; Vikingstad et al. 2000). Motor functions may also be under the control of lateralized mechanisms which may manifest as preference for one side over the other (e.g., handedness, footedness and eyedness) (Brown and Taylor 1988). Laterality is an evolutionarily conserved characteristic which is observed at the populational level and variance in laterality is associated with cognitive and neuropsychiatric disorders including anxiety and major depressive disorder (Koster et al. 2010; Lichtenstein-Vidne et al. 2017).

Lateralization is not a purely human characteristic. Most vertebrate species, including monkeys (Fagot and Vauclair 1991; Hopkins 1994; McGrew and Marchant 1997), rodents (Robison 1981; Rodriguez and Afonso 1993; Rodriguez et al. 1992), birds (Bhagavatula et al. 2014; Franklin and Adams 2010; Gunturkun et al. 1998), fishes (Bibost and Brown 2014; Bisazza and de Santi 2003; Dadda et al. 2010a, b), and some invertebrate species (Anfora et al. 2010; Frasnelli et al. 2012) express brain functional asymmetries. In rodents, for example, several behavioral tasks have been used to assess behavioral asymmetries such as turning rotometers, handedness, choice behavior, T-maze, and Y-maze (Corballis 1986; Pisa and Szechtman 1986; Zimmerberg and Glick 1974). Variability in lateralization exerts a number of fitness benefits at the individual level. For example, lateralization has been associated with maximization of brain processes, enabling individuals to process two tasks simultaneously (Rogers 2000, 2002). Studies have suggested that laterality evolved at the population level to maintain coordination among social groups. Commonality in the direction of lateralization at the group level is as a benefit to group cohesion, providing a potential mechanism, where individuals interact in predictable ways within the group (Rogers 2000).

Taxonomic and evolutionary aspects of brain laterality have been described in fish species, primarily focusing on CNS asymmetries, sensory organs and somatic lateralization, as well as the adaptive role of laterality in nature (de Perera and Braithwaite 2005; Lychakov 2013; Nepomnyashchikh and Izvekov 2006; Vallortigara and Rogers 2005). Behavioral asymmetries have been related to high escape performance (Dadda et al. 2010b), social responses (Reddon and Balshine 2010), and even accelerated learning responses (Andrade et al. 2001), in both fishes and mammals.

Zebrafish (*Danio rerio*) is a versatile vertebrate model system that has been widely used in translational neuropsychiatric research (Fontana et al. 2018; Stewart et al. 2015), and as a model to understand evolutionary aspects of animal cognition (Oliveira 2013; Reale et al. 2007). The last decade has seen an increase in the use of zebrafish to study the mechanisms underlying lateralization (Andersson et al. 2015; Ariyomo and Watt 2013; Barth et al. 2005; Dadda et al. 2010a; Sovrano and Andrew 2006). Larval zebrafish, for example, predominantly use the left-eye when interacting with their own reflection (Sovrano and Andrew 2006). In addition, zebrafish initially use the right hemifield predominantly when interacting with novel objects, but as the object becomes familiar, they switch to the left hemifield (Miklósi et al. 1997). Despite a growing understanding of developmental biology of lateralization in zebrafish (e.g., visual lateralization in learning and memory) (Andersson et al. 2015), very little is known about how spontaneously occurring motor asymmetry impacts on adult zebrafish behavior. Therefore, the principal aims of the present study were: (1) to investigate the existence and incidence of spontaneously occurring motor left–right bias of adult zebrafish in an unconditioned continuous free movement pattern (FMP) Y-maze task and (2) to explore how spontaneously occurring motor left–right bias relates to performance on different behavioral domains, including learning about threat cues in the fear conditioning test, and locomotion and anxiety-related behavior in the novel tank test.

## Materials and methods

### Animals

**Fig. 1:**
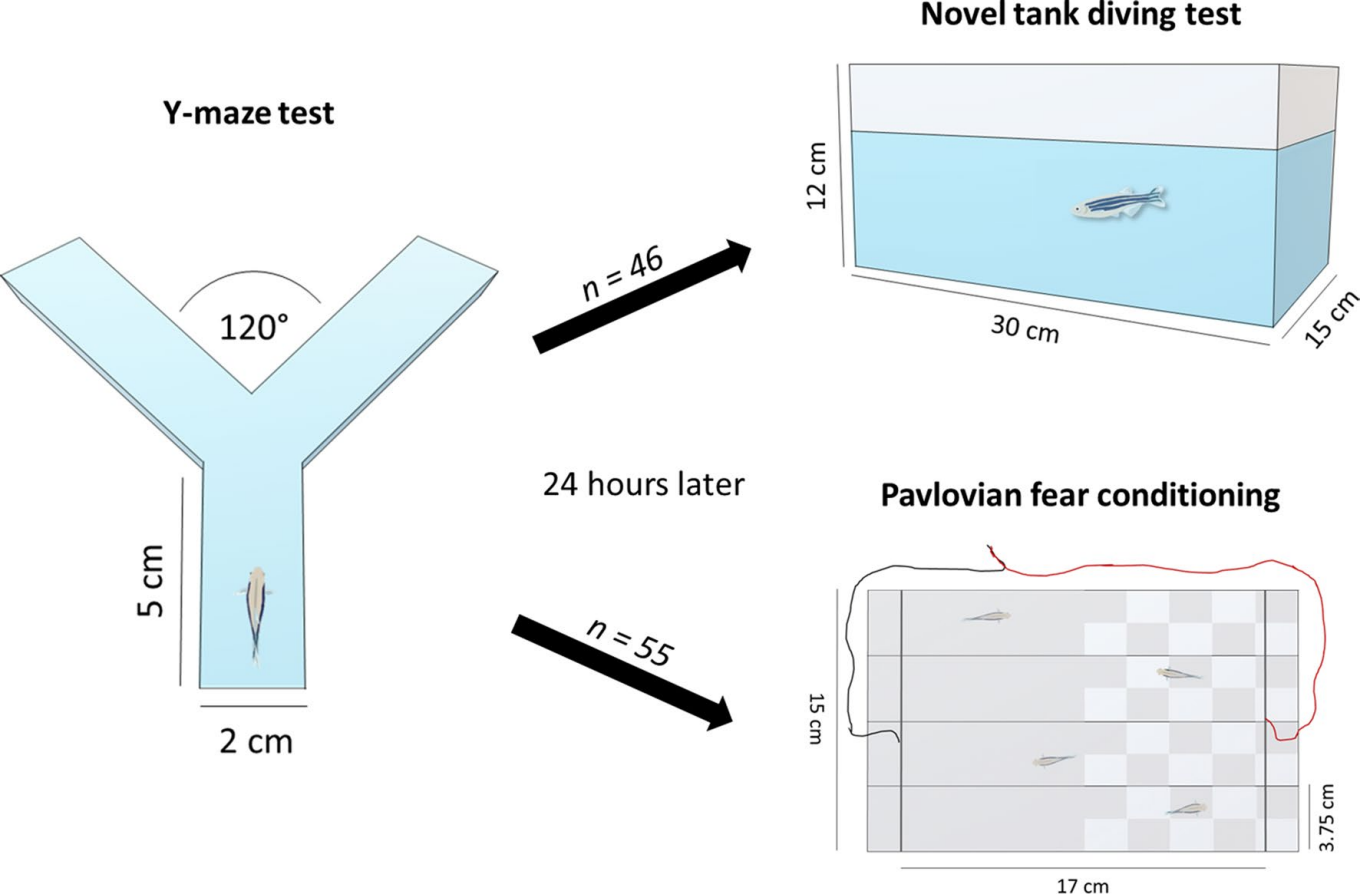
Schematic representation of the experimental design and the behavioral tasks

Adult zebrafish (AB wild type; ~ 50:50 male:female ratio at 3 months of age) were bred in-house and reared in the standard laboratory conditions on a re-circulating system (Aquaneering, USA). Animals were maintained on a 14/10-h light/dark cycle (lights on at 9:00 a.m.), pH 8.4, at ∼ 28.5 °C (± 1 °C) in groups of 20 animals per 2.8 L. Fish were fed three times/day with a mixture of live brine shrimp and flake food, except at weekends when they were fed once a day. Animals were tested in the FMP Y-maze apparatus and then pair-housed for 24 h prior to analysis of shock avoidance or tank diving, to reduce stress from multiple handling in a single day (see Fig. 1). During the pair-housing period, animals had shared water system and visual contact through transparent partition, the pair-housing system is used to separate animals for further identification and reduce the stress induced by social isolation (Parker et al. 2012). After behavioral tests, all animals were euthanized using 2-phenoxyethanol from Aqua-Sed (Aqua-Sed™, Vetark, Winchester, UK). All experiments were carried out following approval from the University of Portsmouth Animal Welfare and Ethical Review Board, and under license from the UK Home Office [Animals (Scientific Procedures) Act, 1986] [PPL: P9D87106F].

### Free movement pattern Y-maze task

Y-maze spontaneous alternation tasks have previously been used to assess left- and right-biased responses (Barnard et al. 2016; Castellano et al. 1987; Frasnelli 2013; Rodriguez et al. 1992). Here, we used a novel version of the Y-maze developed in our lab. In the FMP Y-maze task, fishes behavior patterns were recorded for 1 h. Choices at each fork in the maze were binary and could either be ‘left’ or ‘right’, and thus represent a series of non-discrete choice “trials” within the continuous search task. Choices were quantified for each fish in terms of continuously overlapping series of four choices (tetragrams; llll, lllr, llrl, [etc.]…, rrrr) (Cleal and Parker 2018; Gross et al. 2011). Analysis was normalized by calculating frequency of each tetragram as a proportion of the total number of turns. For assessing fishes’ behavioral lateralization, the number of turns to the right or left was calculated. To calculate index of bias, choices (in 10-min time bins) were entered into the following formula: ${\text{Bias}}\;{\text{percentage}} = \frac{{{\text{number}}\;{\text{of}}\;{\text{turns}}\;L\;{\text{or}}\;R \times 100}}{{{\text{total}}\;{\text{number}}\;{\text{of}}\;{\text{turns}}}}$. Following this calculation, the mean and coefficient of variation (CV) for right and left bias were calculated across the 10-min time bins to assess the consistency of data across the entire 1-h test period.

One-hundred and three adult zebrafish were used for assessing FMP Y-maze performance and right–left bias. Required sample size was calculated a priori following pilot tests, in which we examined the relative frequency of biased animals (showing > 60% side bias) to unbiased [effect size (*d*) = 0.3, power = 0.8, alpha = 0.05]. Each fish was used only once, and we carried out three complete independent batches on different days. Behavioral tests were performed between 10 a.m. and 4 p.m. using the Zantiks [AD] fully automated behavioral testing environment (Zantiks Ltd., Cambridge, UK; Brock et al. 2017). The Zantiks AD system was fully controlled via a web-enabled device during behavioral training. The test tank consisted of two identical white plastic Y-mazes, each with three identical arms (5 cm length × 2 cm width; arms at a 120° angle from each other) and a transparent base (Fig. 1). The tank in which the maze was located was filled with 3 L of aquarium water. Ambient light (44 lx) allowed some visibility in the maze, but no explicit intra-maze cues were added, to ensure that each fishes’ perceived location remained ambiguous, regardless of its relative position in the maze. Behavioral lateralization was considered when the fish carried out > 60% of right or left turns during the 1-h test. To assess zebrafish search strategies, we used the number of alternations (rlrl + lrlr) and repetitions (rrrr + llll) as proportion of total number of turns which are highly expressed through 1 h and are commonly linked to animals’ exploratory activity (Cleal and Parker 2018; Gross et al. 2011). Relative alternations have been suggested to be a measure of working memory, with a relative reduction the number of alternations using the tetragram methodology having been observed following developmental alcohol exposure (Cleal and Parker 2018), while relative repetitions are suggested to be a measure of response perseveration (Gross et al. 2011).

### Pavlovian fear conditioning

The Pavlovian inhibitory avoidance paradigm is a valid method widely used to explore mechanisms underlying fear avoidance learning responses in zebrafish (Amorim et al. 2017; Manuel et al. 2014, 2015; Ng et al. 2012). Twenty-four hours after the completion of the FMP Y-maze task, fish (*n* = 55) were tested on a Pavlovian fear conditioning procedure for 1 h. The fear conditioning response was based on previous work (Cleal and Parker 2018; Valente et al. 2012). Fish were individually placed in one of the four lanes of a tank (25 cm length × 15 cm, 1 L of water in each tank) of the Zantiks (Zantiks Ltd., Cambridge, UK) AD system (Brock et al. 2017) (Fig. 1). Briefly, fishes were habituated for 30 min in the test environment, during which time, half of the arena had a check pattern on the base and the other half had a grey color on the base (alternating between sides of the tank every 5 min). Images were displayed on a screen in the base of the AD unit, on which the test tank sits. Following 30-min habituation, 10-min baseline data were collected to assess preference for the stimuli. Following the baseline was the conditioning phase, which consisted of a conditioned stimulus (CS+ ; full screen of “check” or “grey” [half CS+ in “check” and CS+ in “grey”]) presented for 1.5 s and followed by a brief mild shock (9 V DC, 80 ms; unconditioned stimulus (US). After this, an 8.5-s inter-trial interval (ITI) of the non-CS (CS−) exemplar was presented at the bottom of the tank. The CS+/US was presented nine times. Finally, avoidance of CS+ was assessed by presenting CS+ and CS− simultaneously for 1 min at either end of the tank (switching positions after 30 s) and calculating the time spent in the vicinity of CS+, and comparing this to baseline preference.

### Novel tank diving and thigmotaxis responses

The novel tank test measures locomotor and anxiety-like phenotypes in zebrafish, and is highly sensitive both to anxiolytic and anxiogenic drugs (Egan et al. 2009; Kalueff et al. 2013; Levin et al. 2007; Maximino et al. 2010; Mezzomo et al. 2016; Wong et al. 2010). Twenty-four hours after the FMP Y-maze test, animals (*n* = 46) were placed individually in a novel tank (30 cm length × 15 cm height × 12 cm width) containing 4 L of aquarium water. Behavioral activity was recorded using two webcams (front and top view, see Fig. 1b) for 5 min to analyze thigmotaxis and diving response (Egan et al. 2009; Parker et al. 2012; Rosemberg et al. 2012). Behaviors were measured using an automated video-tracking software (EthoVision, Noldus Information Technology Inc., Leesburg, VA—USA) at a rate of 60 frames/s. Thigmotaxis (time spent in proximity to the edge/sides) was analyzed through behavioral tracking obtained by the top view camera. Meanwhile, for a detailed evaluation of vertical activity, the tank was separated in three virtual areas (bottom, middle, and top) and the following endpoints were measured: total distance traveled, time spent in each third of the tank, and immobility.

### Randomization and blinding

All behavioral testing was carried out in a fully randomized order, choosing fish at random from one of the ten housing tanks for testing. Fish were screened for left–right bias in the FMP Y-maze first, but analysis was not carried out prior to subsequent behavioral testing to avoid bias. Subsequent to the FMP Y-maze screening, fish were pair-housed and issued a subject ID, allowing all testing to be carried out in a fully blinded manner. Once all data were collected and screened for extreme outliers (e.g., fish freezing and returning values of ‘0’ for behavioral parameters indicating non-engagement), the bias was revealed, and data analyzed in full. Two animals were excluded in the FMP Y-maze task and two in the novel tank due to poor engagement with the task (freezing and displaying no measurable behavioral patterns).

### Data processing and statistical analysis

Raw data from the FMP Y-maze task was in the form of arm entries. To analyze the data according to left and right turns in 10-min time bins, raw data were processed using a custom-written R (www.r-project.org) script (available from: https://github.com/thejamesclay/ZANTIKS_YMaze_Analysis_Script). Subsequently, data were analyzed in IBM SPSS^®^ Statistics and the results were expressed as mean ± standard error of the mean (S.E.M), to assess whether there were any effects of ‘bias’ (> 60% preference for left/right) on total turns, alternations (lrlr + rlrl) or repetitions (rrrr + llll). Alternations and repetitions were analyzed using generalized linear models (Poisson distribution and log link), with laterality (three levels—left bias, right bias, and non-bias) and time (six levels—10-min time bins across 1 h) as the fixed factors, and ID as a random effect (to account for non-independence of replicates). To analyze novel tank responses, we used either one- or two-way ANOVAs, with bias and time spent in different tank zones fixed factors (novel tank) or bias only as a fixed factor for thigmotaxis, immobility, and distance traveled. In addition, left–right-bias effects on the shock-avoidance test were assessed using two-way repeated measures ANOVA with ‘bias’ (left vs right vs neutral) and conditioning (pre vs. post) as factors, and preference for conditioned stimulus as the dependent variable. Newman–Keuls test was used as post hoc analysis, and results were considered significant when *p* ≤ 0.05.

## Results

### Left–right-bias profile in the FMP Y-maze test

**Fig. 2:**
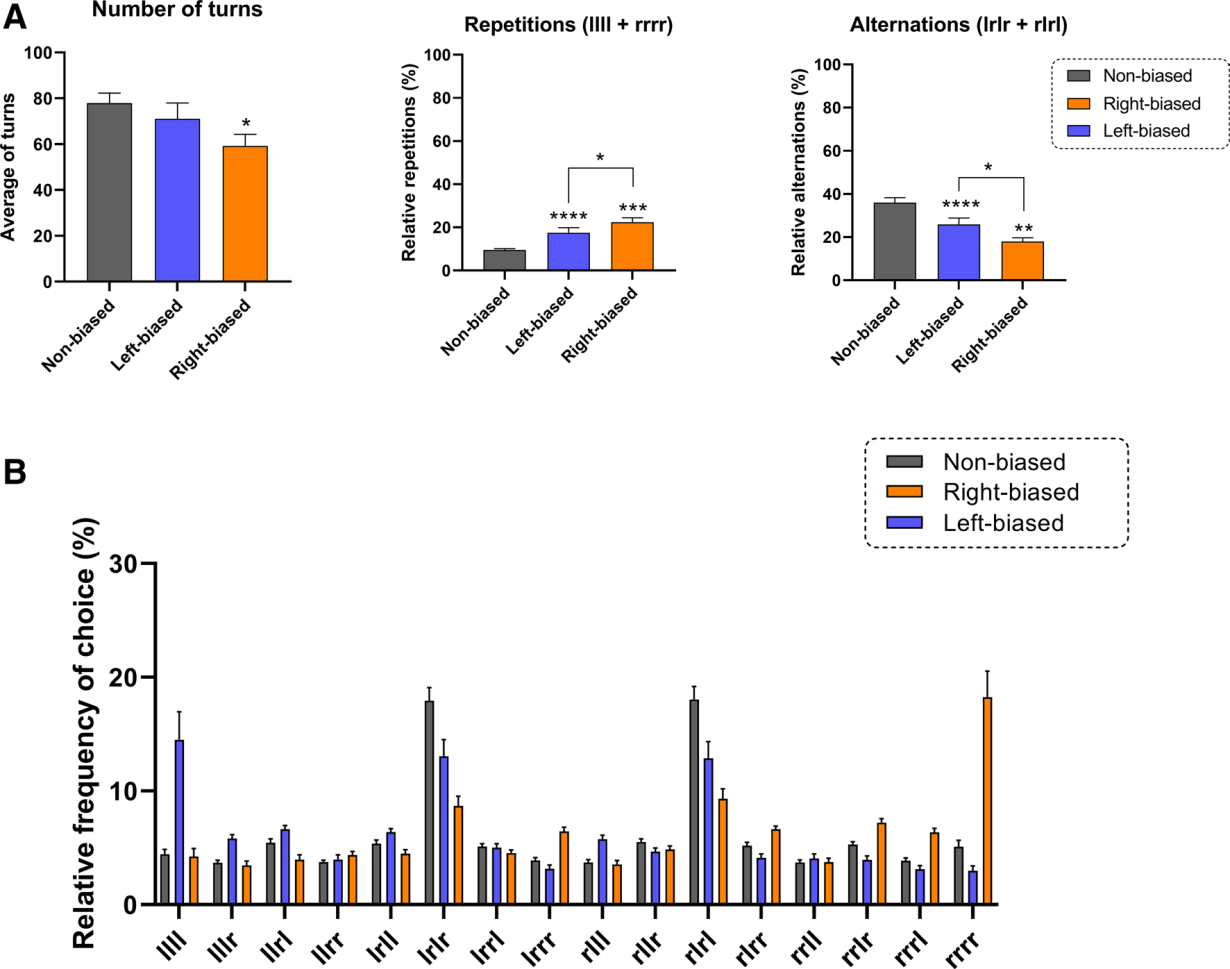
Effects of left- and right bias in zebrafish on the Y-maze test. **a** Laterality affects total number of turns, repetitions and alternation of adult zebrafish. **b** Y-maze tetragrams showing the behavioral phenotype of biased and non-biased animals considering the relative frequency of choice (tetragram frequency of choice × 100/total number of turns). Data were represented as mean ± S.E.M. and analyzed by linear mixed effects, followed by Tukey’s multiple comparison test. Asterisks indicates statistical differences compared to non-biased group or between biased groups (*p < 0.05, **p < 0.01, ***p < 0.001 and ****p < 0.0001, *n* = 47 non-biased, *n* = 28 left-biased and *n* = 28 right-biased group)

Zebrafish showed behavioral lateralization in the FMP Y-maze (right biased 27.18%, left-biased 27.18% and non-biased 45.63%). First, we confirmed that behavioral laterality was consistent across 1 h (analyzed in 10-min time bins), by calculating the coefficient of variation (CV) for the left and right-turn preferences for the non-biased (left CV 19.28 ± 2.52 and right CV 21.05 ± 2.95), left-biased (left CV 30.40 ± 3.85 and right CV 21.7 ± 3.61), and right biased (left CV 27.23 ± 5.52 and right CV 25.95 ± 2.31) groups. Figure 2 displays the FMP Y-maze data. A significant bias effect was observed for number of turns [*F*_(2, 601)_ = 13.115; *p* < 0.0001], repetitions [*F*_(2, 601)_ = 39.696; *p* < 0.0001], and alternations [*F*_(2, 601)_ = 45.437; *p* <  0.0001]. A time effect (data not shown) was also observed for number of turns [*F*_(5, 601)_ = 9.769; *p* < 0.0001], repetitions [*F*_(5, 601)_ = 3.242; *p* = 0.007], and alternations [*F*_(5, 601)_ = 3.801; *p* = 0.002]. In addition, a significant interaction effect (bias × time) was observed for repetitions [*F*_(10, 601)_ = 2.504; *p* = 0.006] and alternations [*F*_(10, 601)_ = 2.390; *p* = 0.009]. Bias, to the left or the right, significantly increased the number of repetitions (*p* < 0.0001 for right bias and *p* < 0.001 for left bias) and decreased the percentage of alternations (*p* < 0.0001 for right bias and *p* < 0.005 for left bias) compared to non-biased animals. In addition, right-biased fish decreased the number of turns (*p* < 0.05) compared to non-biased fish. Differences were not just observed between biased vs. non-biased groups, but also between bias groups, with right-biased fish displaying a significant increase in repetitions (*p* < 0.05) and decrease in alternations (*p* < 0.05) compared to left-biased fish (Fig. 2a). The behavioral profile of biased and non-biased fish is displayed in terms of tetragrams (Fig. 2b), where the relatively high number of llll and rrrr configurations can be observed for left- and right-biased animals, respectively.

### Short-term avoidance memory and novelty response of biased animals

**Fig. 3:**
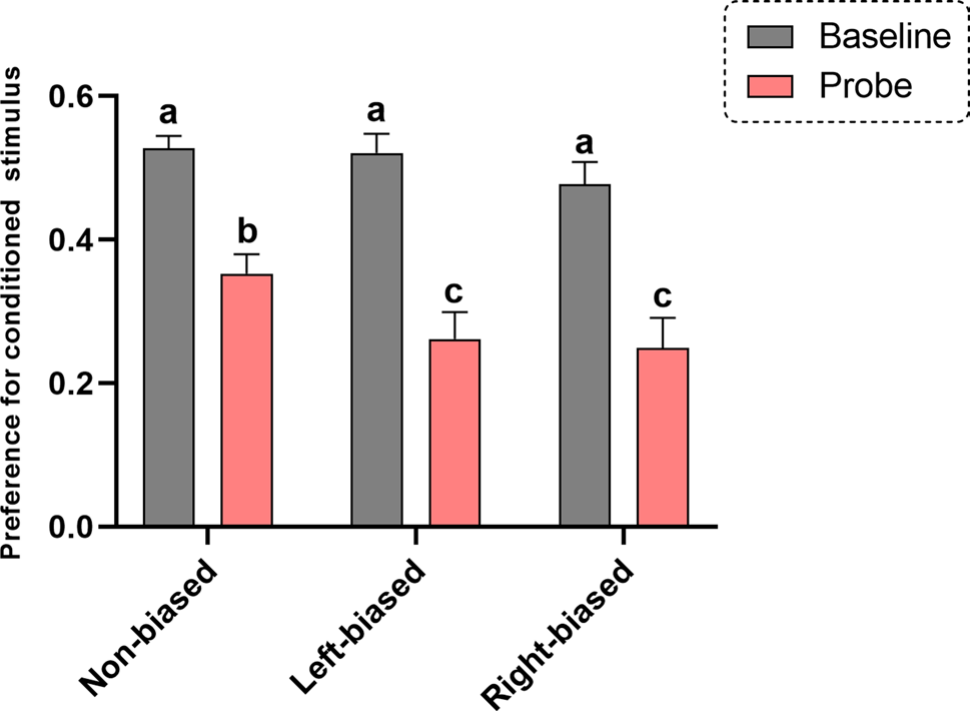
Left- and right bias are related to fear avoidance learning responses in adult zebrafish. Data were represented as mean ± S.E.M. and analyzed by two-way RM ANOVA, followed by Tukey’s multiple comparison test. Different letters indicate significant differences between groups (*p* < 0.05; *n* = 25 non-biased, *n* = 17 left-biased and *n* = 13 right-biased group)

**Fig. 4:**
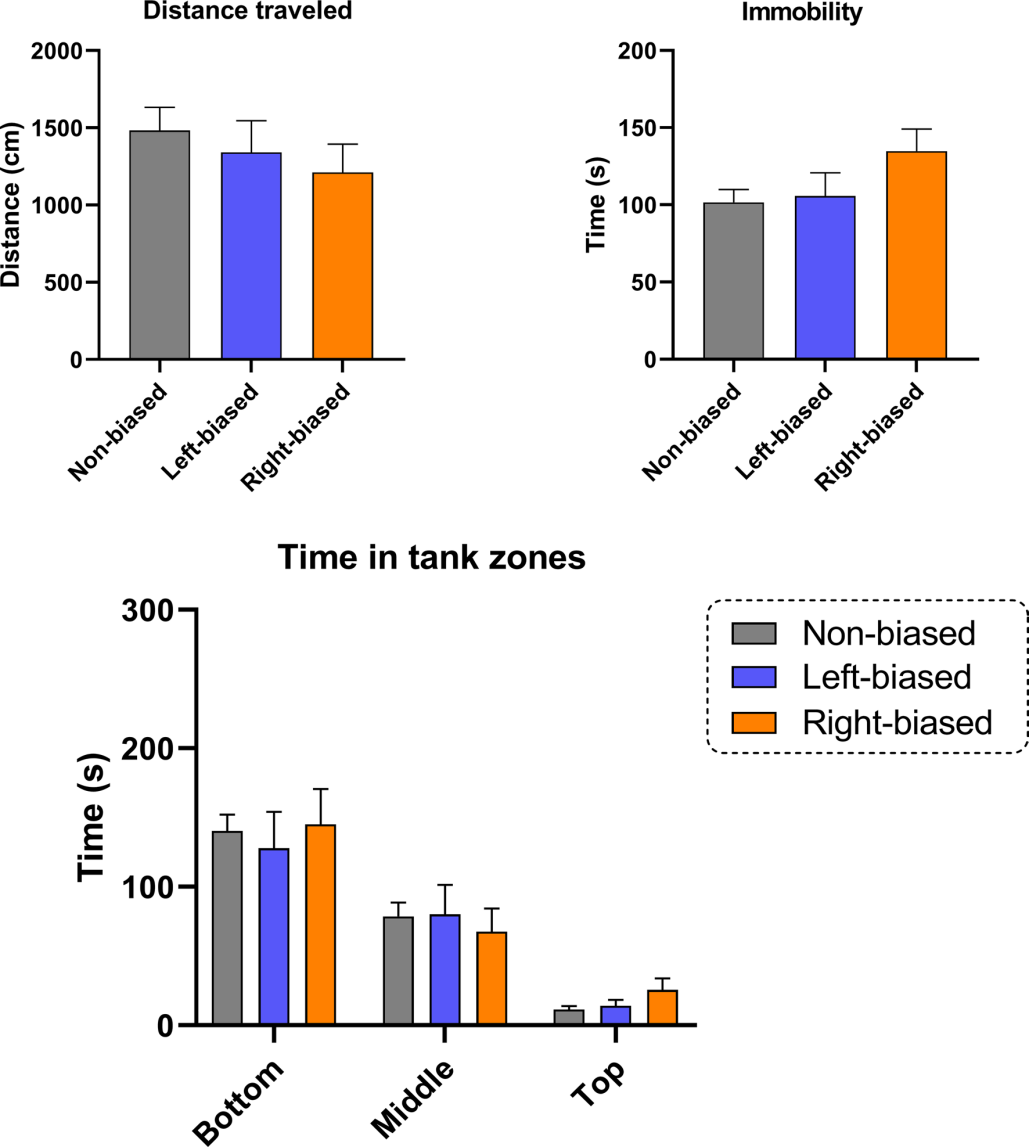
Behavioral laterality is not related to locomotor or anxiety-related phenotypes in adult zebrafish. Data were represented as mean ± S.E.M. and analyzed by one- or two-way ANOVA (*n* = 22 non-biased, *n* = 11 left-biased and *n* = 15 right-biased group)

**Fig. 5:**
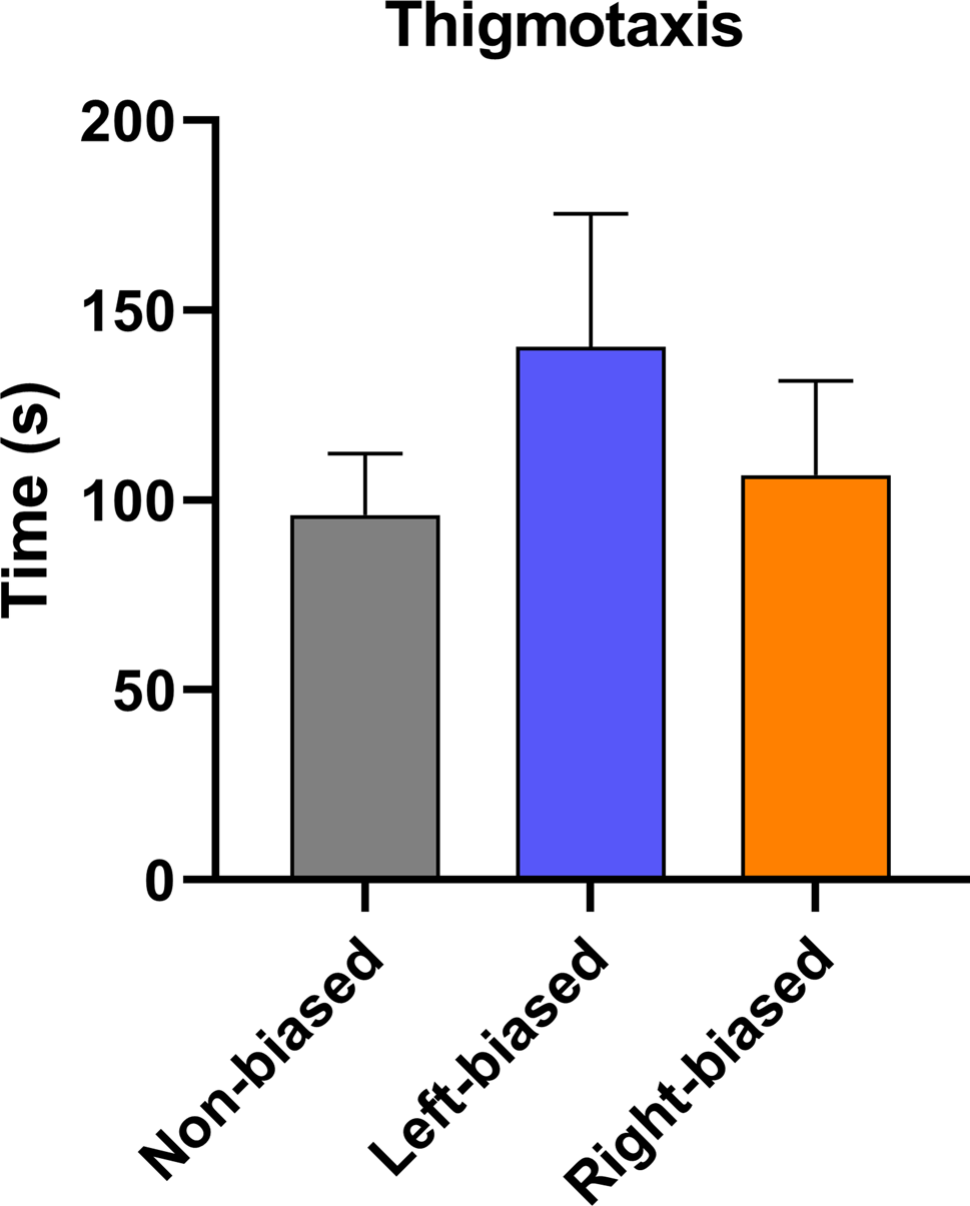
Left- and right bias do not change thigmotaxis in adult zebrafish. Data were represented as mean ± S.E.M. and analyzed by one-way ANOVA (*n* = 20 non-biased, *n* = 11 left-biased and *n* = 15 right-biased group)

Although no interaction effect bias vs. shock [*F*_(2, 98)_ = 1.259; *p* = 0.312] was observed for the Pavlovian responses, a significant effect for bias [*F*_(2, 98)_ = 3.128; *p* = 0.035] and conditioning (probe vs. baseline) [*F*_(1, 98)_ = 79.47; *p* < 0001] effect was revealed. ANOVA analyses are often underpowered and, therefore, unable to detect the significance of interaction terms (Wahlsten 1990). Therefore, post hoc analysis was performed to specifically analyze the effects between groups. In general, all biased (*p* < 0.0001 for right- and left bias) and non-biased (*p* < 0.0001) animals had a decreased time spent in the preference for conditioned stimulus (probe vs. baseline). However, both left- and right-biased animals (*p* < 0.05) spent significantly less time in the conditioned area during the probe trial compared to non-biased group (Fig. 3), despite no significant differences in their baseline preferences, suggesting a stronger response to the aversive stimulus. No significant effect was observed for bias in all novel tank diving test-related parameters, including distance traveled [*F*_(2, 43)_ = 0.683; *p* = 0.510], immobility [*F*_(2, 43)_ = 2.348; *p* = 0.107], time in tank zones time [*F*_(2, 129)_ = 0.084; *p* = 0.918] (Fig. 4), and thigmotaxis [*F*_(2, 43)_ = 1.289; *p* = 0.286] (Fig. 5).

## Discussion

In this study, we evaluated spontaneously occurring motor left–right bias in adult zebrafish using the continuous unconditioned FMP Y-maze task, and evaluated the predictive validity of spontaneous behavioral laterality on both unconditioned and conditioned measures of fear and anxiety. We showed, for the first time, that the zebrafish presents spontaneous behavioral laterality in the FMP Y-maze test when analyzed using 16 overlapping tetragrams to evaluate exploration strategy, suggesting that the protocol may be useful for screening this species for behavioral asymmetry. Second, we found that behaviorally lateralized animals show decreased alternations and increased repetitions patterns in the FMP Y-maze compared to non-biased animals. This suggests that search strategies used by spontaneously biased individuals differ from that used by non-biased fish and is quite likely directly due to the lateralization. Third, we observed that behavioral asymmetry predicts increased aversive responses in a Pavlovian fear conditioning protocol, but did not predict measures of unconditioned anxiety (novel tank test, thigmotaxis). Collectively, these data suggest, contrary to theories that laterality bias in fish is related to increased stress-reactivity, that increased behavioral laterality may be related to increased cue reactivity, particularly in relation to aversive cues. This has connotations for translational models of human disorders of affective state, in which heightened attention to threat-related cues is observed (Lichtenstein-Vidne et al. 2017) and in which variations in brain and behavioral laterality are thought to be risk factors (Bruder et al. 2016).

Left–right asymmetries in behavioral protocols including the T-maze and Y-maze have been widely utilized in rodents (Andrade et al. 2001; Nakagawa et al. 2004; Rodriguez et al. 1992). Here, for the first time, we observed that approximately a quarter of zebrafish present substantial natural left- (27.18%) or right- (27.18%) locomotor lateralization, with the remaining 45.63% of animals showing stochastic patterns of left/right. These data are somewhat at odds from observed bias in other models, in which there is a high number of right-biased animals (52.8%) and low numbers of left- (22.2%) and non-biased responses (25%) for rodents (Andrade et al. 2001). Using an open field detour test (Chivers et al. 2016) showed that wild-caught fish, yellow-and-blueback fusiliers (*Caesio teres*) present a spontaneous increased bias for right turns (65%) and decreased bias for left turns (24%), with no left/right bias representing the least common phenotype (11%). However, they also observed that the strength of the behavioral lateralization was increased depending on predation pressure and that right-biased fish, in general, displayed better escape performance as compared to left-biased and non-biased individuals (Chivers et al. 2016).

Studies focusing on the evolutionary aspects of lateralization suggest that behavioral asymmetries exist at population level, because individually asymmetrical organisms coordinate their behavior with other asymmetrical organisms (Frasnelli 2013). For example, right-biased animals showed increased escape responses (Chivers et al. 2016) and increased right-eye use when attacking the mirror image aggression (Bisazza and de Santi 2003). In invertebrates, laterality has also been consistent with the vertebrate findings, where eusocial ants show motor left bias when exploring unfamiliar nest sites (Hunt et al. 2014), and eusocial honeybees have olfactory asymmetries that predict learning and recall of memory (Frasnelli et al. 2010; Rigosi et al. 2011; Rogers and Vallortigara 2008).

We observed that behavioral laterality has an important role in FMP Y-maze performance, where left–right-biased animals presented an increase of repetition behavior and decrease of alternation, in particular in the left-biased animals. Previous studies (Cleal and Parker 2018; Gross et al. 2011) and continuing work in our laboratory, in conjunction with rodent T- and Y-maze data, suggest that typical spontaneous search strategy employed is to use a relatively high level of alternations. Alternations have been directly associated with functionally distinct search patterns, where the seeking for change and novelty may have a role in their exploratory profiles (Kool et al. 2010). Rodriguez et al. (1992) described decreases in pure alternations and increases in pure repetition behavior in lateralized animals, and demonstrated that both stress and over-training decrease the alternation/repetition ratio through the promotion of an increase of biased responses. Although the dominant strategy, alternations and, to a far lesser extent, repetitions, are punctuated with other more stochastic patterns, as indicated in Fig. 2b showing the relative frequency of each of the 16 tetragrams throughout the 1-h trial. We have suggested before that the dominant search strategies used in the FMP Y-maze task are associated with motor working memory (alternations) or perseveration (repetitions) (Cleal and Parker 2018). This theory of what underlies the dominant strategies would suggest that lateralized zebrafish are more strategically perseverative, as opposed to using working memory. However, further work would have to be carried out to determine the mechanisms underlying alternations and repetitions in FMP Y-maze performance. Here, we confirmed that both alternations and repetitions remain as a highly reliable behavioral pattern that is conserved across species, and that variations in FMP Y-maze may be a fruitful direction for more detailed analysis in the future (Ghafouri et al. 2016; Lewis et al. 2017; Pickering et al. 2015).

In agreement with the previous studies (Andrade et al. 2001), we showed that locomotor lateralization is associated with increased learning in a Pavlovian fear conditioning protocol. Studies using other fish species showed that lateralized animals have a better response in cognitive tasks such as spatial reorientation (Sovrano et al. 2005), and left-eye bias is related to faster learning in conditioning tasks (Bibost and Brown 2014). In general, the most predominant theory of how left–right bias affects learning and cognitive processing relates to a hypothesized increased stress-reactivity in lateralized animals (Carlson and Glick 1989; Neveu 1996; Westergaard et al. 2001). Interindividual differences in laterality have been shown to covary with, or predict, individual differences in stress-reactivity and susceptibility to stress-related pathology (Byrnes et al. 2016; Carlson and Glick 1989; Fride and Weinstock 1989; Ocklenburg et al. 2016). Here, we tested the hypothesis that left- and right-biased animals would differ in measures of stress-reactivity and anxiety-like phenotypes (Blaser and Rosemberg 2012; Egan et al. 2009; Parker et al. 2012). We found no significant differences in lateralized animals in our measures of anxiety, suggesting that the observed differences in behavioral phenotypes observed in the FMP Y-maze and Pavlovian avoidance learning seems to not be related to stress-reactivity responses per se. Instead, our data may suggest that the lateralized fish are more reactive to stress-related cues. This would explain the increased performance in the Pavlovian fear conditioning, as well as the fact that there were no differences in measures of general anxiety. In addition, if the lateralized fish perceived the Y-maze as a more threatening or ‘negative’ environment, this may explain their change in search strategy: the role of stress in perseveration is well established (Ridley 1994).

There are several theories regarding the mechanisms underlying behavioral laterality in simple maze tasks. Diaz Palarea et al. (1987) were the first to report that left–right-biased animals, as assessed via spatial asymmetry in a T-maze, had alterations in dopaminergic (DA) signaling. In addition, apomorphine (non-specific DA receptor agonist) and 6-hydroxydopamine lesions alters behavioral laterality of animals in the T-maze test (Castellano et al. 1987) and Y-maze (Nakagawa et al. 2004), confirming the involvement of DA system in behavioral asymmetry. DA receptors are strongly implicated in emotional learning and recall of emotionally relevant events in rats. For example, activation of D4-receptors in the medial pre-frontal cortex potentiates fear-associated memory formation, but has no impact on recall (Lauzon et al. 2009; Laviolette et al. 2005), whereas activation of D1-like receptors blocks recalls of previously learned fear-associated memories, but has no impact on learning (Lauzon et al. 2009), suggesting a double dissociation of function. Interestingly, the serotonergic (5-HT) system has also been shown to have an important role in mediating individual differences in anxiety-like responses and locomotor activity in zebrafish and exerts a minor modulatory role of the DA system (Tran et al. 2016). Both behavioral laterality and aversive memory is mostly associated with modulatory action of the DA system, but the 5-HT system has a major role modulating zebrafish responses to novelty. The precise mechanisms of how behavioral laterality modulates neuropsychiatric conditions are yet to be firmly established, and further studies are required to better understand the mechanisms in which behavioral laterality modulates aversive memory in zebrafish. Our data have shown that the FMP Y-maze may be a very useful tool for carrying out such research and should be exploited in the future.

## Conclusion

We showed for the first time that zebrafish exhibit spontaneously occurring motor lateralization which can influence aversive learning responses. We also found that biased animals show an altered exploratory search strategy in the FMP Y-maze, including higher repetitions and lower alternations. Coupled with a lack of observed differences between lateralized and non-lateralized animals in unconditioned tests of anxiety, our data suggest that lateralized zebrafish may show heightened reactivity to fear related cues. These results have important connotations for translational models of depression and anxiety, particularly in the light of well-established links between laterality and anxiety/depression in humans. Finally, because biased animals present different behavioral performances in the FMP Y-maze and Pavlovian fear conditioning protocols, left- and right- preference should be considered when working with zebrafish behavior, particularly to control variability in performance on more complex tasks.
